# Stacking and Analysis of Melamine in Milk Products with Acetonitrile-Salt Stacking Technique in Capillary Electrophoresis

**DOI:** 10.1155/2014/212697

**Published:** 2014-08-18

**Authors:** Yu Kong, Chong Wei, Zhanwu Hou, Zilong Wang, Jiaqiang Yuan, Jiang Yu, Yongxi Zhao, Yuhai Tang, Meili Gao

**Affiliations:** ^1^Institute of Mitochondrial Biology and Medicine, The Key Laboratory of Biomedical Information Engineering of Ministry of Education, Department of Bioengineering, School of Life Science and Technology, Xi'an Jiaotong University, Xi'an 710049, China; ^2^Department of Criminal and Inspectoral, Public Security Bureau of Han Zhong City, Shaanxi, China

## Abstract

Melamine was measured in real milk products with capillary electrophoresis (CE) based on acetonitrile-salt stacking (ASS) method. Real milk samples were deproteinized with acetonitrile at a final concentration of 60% (v/v) and then injected hydrodynamically at 50 mBar for 40.0 s. The optimized buffer contains 80.0 mmol/L pH 2.8 phosphates. Melamine could be detected within 20.0 min at +10 kV with a low limit of detection (LOD) of 0.03 *μ*mol/L. Satisfactory reproducibility (inter- and intraday RSD% both for migration time and peak area was lower than 5.0%) and a wide linearity range of 0.05 *μ*mol/L ~ 10.0 *μ*mol/L were achieved. The proposed method was suitable for routine assay of MEL in real milk samples that was subjected to a simple treatment step.

## 1. Introduction

Melamine (MEL) in animal foods and milk products had caused serious accidents, as its metabolism, melamine cyanurate, was easily crystallized in organs and was tending to cause kidney stone and damage [1–3]. Therefore, establishing a powerful rapid sensitive analytical method for measuring low concentration of MEL in kinds of milk products would benefit the routine assay of the related products.

Several analytical methods, such as gas chromatography-mass spectrometry (GC-MS) [4], high performance liquid chromatography (HPLC) [5–7] gold nanoparticles [8], colloidal selenium immunoassay [9], and Terahertz time-domain spectroscopy [10] had been reported. However, many methods depended on complicated sample treatments. As in GC-MS methods, the sample derivation was strongly demanded as MEL contains special label group [4]; as in HPLC methods, liquid-liquid, liquid-solid extraction procedure was needed to cleanse the sample and perform better separation results. In some cases, in order to lower the limit of detection (LOD) or obtain detailed information of the analytes, special detectors, such as mass spectrum (MS) [5], fluorescence detection [6], surface enhanced raman scattering [8], electrochemical approach [11], and online solid-phase extraction [7], were also used. However, some of these detectors were uncommon or had special demands. For example, in MS detecting methods, salt-free samples were required.

Capillary electrophoresis had advantages on separating complicated samples and had been applied to determine MEL [12–14]. Moreover, CE online stacking methods, which could easily perform enrichment of low-concentration analytes and achieved lower LODs, had also been used for assay of MEL in real samples [15–19]. Jin et al. [16] proposed several stacking methods, such as including large-volume sample stacking-sweeping and selective exhaustive injection-sweeping (SEI-S, in either cation or anion mode), for determination of melamine and its derivatives in liquid milk. LOD of MEL was around 0.01 ng/mL, however, it's difficult to achieve better sensitivities for all derivatives in one mode. Li et al. [17] combined cation selective exhaustive injection (CSEI) with sweeping-MEKC method. In their study, the LOD was 23.4 pg/mL for MEL; however, the recovery of MEL was unsatisfied (ca. 74–83%). Wu et al. [18] developed a MEKC sweeping method for MEL, face-centred cube central composite design was used for optimization of conditions, and the LOD of MEL could reach 5 ng/mL with satisfied recovery within 13 min.

In our works, acetonitrile-salt stacking (ASS) [20–25] was used for detecting MEL under cation mode in milk products. The factors that influenced both stacking and separation, such as buffer pH, sample acetonitrile concentration, sample salt concentration, injection time, buffer concentration, stacking and separation voltage, were carefully investigated and optimized.

## 2. Experimental

### 2.1. Chemicals

Melamine was purchased from Sigma (St. Louis, MO, USA). Phosphoric acid, sodium dihydrogen phosphate, disodium hydrogen phosphate, sodium phosphate, sodium chloride, and acetonitrile (HPLC-grade agents) were purchased from Tianjin chemical reagent company (Tianjin, China). Water used throughout was purified by OLST M6 (Ao Lian Co, Xian, China) with a conductivity of 18.2 *Ω* · cm and all the solutions and samples were made daily and filtered through 0.22 *μ*m filter.

### 2.2. Instrumentation

Experiments were performed on Agilent 3D CE Capillary electrophoresis system (Agilent, USA) equipped with a diode array detector (DAD) and a temperature controller (15~60 ± 0.1°C). Instrument control and data analysis were carried out by Agilent Chem station (Rev.A.09.03, Agilent, USA) on a personal computer. For pH measurements, a pH meter (PB-10, Sartorius, Germany) calibrated with a precision of 0.01 pH unit was employed. Centrifugation was performed on a 5810R (Eppendorf, Germany). All the CE experiments were performed on a fused silica capillary (Yongnian photoconductive fibre factory, Hebei, China).

### 2.3. Preparation of Milk Samples and Standard Samples

The milk samples, obtained from local markets, were deproteinized with acetonitrile at the rate of 6 : 4 (acetonitrile: milk, v/v) or as specified. Then, the treated samples were centrifuged for 15 min at 14,200 rpm to eliminate the precipitates. All supernatants were assayed after being added with 100 mmol/L NaCl and filtration.

Standard stock solutions of melamine (8 mmol/L) were prepared quantitatively with water and stored at 4°C until usage. All working standard samples contained 8 mmol/L phosphate buffer and required concentration of sodium chloride and 60% acetonitrile (or as specified) and ~10 *μ*mol/L MEL (final concentration).

### 2.4. Electrophoresis Procedure

All the separations were performed on a fused-silica capillary (75 *μ*m I.D., effective length of 39 cm, and total length of 50 cm) at 20°C with a constant separation voltage of +10.0 kV. For the first usage, the capillary was activated with 1.0 mol/L NaOH for 1 hour. The stacking and separation buffer consisted of 80 mmol/L phosphate buffer (pH 2.8). The samples were injected hydrodynamically at +50 mBar for 40.0 s (about 20% of the capillary efficient length) or as specified. Before each run, the capillary was rinsed with 0.1 mol/L NaOH for 3 min at 1.4 × 10^3^ mBar.

## 3. Results and Discussion

As the ASS method stacked samples via transient pseudoisotachophoresis way and separated samples via capillary zone electrophoresis mode, several factors that affected stacking, such as sample matrix, voltage, injection time, and separation, such as buffer pH, were carefully studied. In general, MEL was positively charged under the selected condition. It was stacked based on its difference of migrating velocity between sample zone (faster) and outlet buffer zone (slower).

### 3.1. Influence of Buffer pH and Concentration on Separation

**Figure 1:**
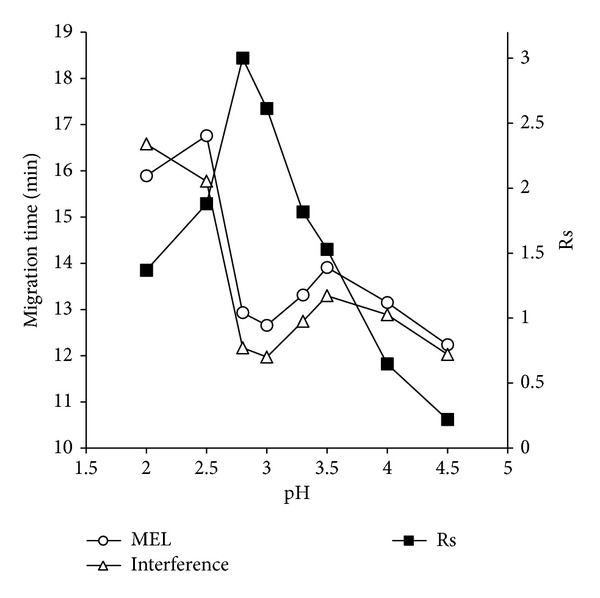
Influence of pH on migration times and Rs values.

Buffer pH was one of most important factors that would influence separation and stacking. In this section, a pH range of 2.0~5.0 was firstly studied, as the pKa values of MEL were about 5.0; it could only be positively charged when buffer pH was lower than 5.0. There was an interferent compound in blank milk sample (contained no MEL, data not shown) which had similar migration time as MEL. Regarding this, the optimization of pH was mainly based on the resolution (Rs) between MEL and interferent as well as their migration times. The influence of pH on Rs and migration times were shown in Figure 1. It indicated that, at the lower pH (<2.5), the electroosmotic flow (EOF) was nearly zero, and MEL was strongly and positively charged and migrated to the detecting window by itself; when the pH became higher, the silanol started to dissociate and, as a result, the EOF slightly increased and migration times dropped; pH 3.5 had the slight longer migration time compared with pH 3.0, which may be caused by the faster decreasement of effective positive charges of MEL compared with the slower increasement of EOF. In addition, a peak order change was also observed in pH range of 2.0~2.5, which was similar to our CZE separation works (data not shown). Finally, based on the curve of Rs in Figure 1, pH 2.8, which had the best Rs value and relative shorter migration time, was chosen for further usage.

**Figure 2:**
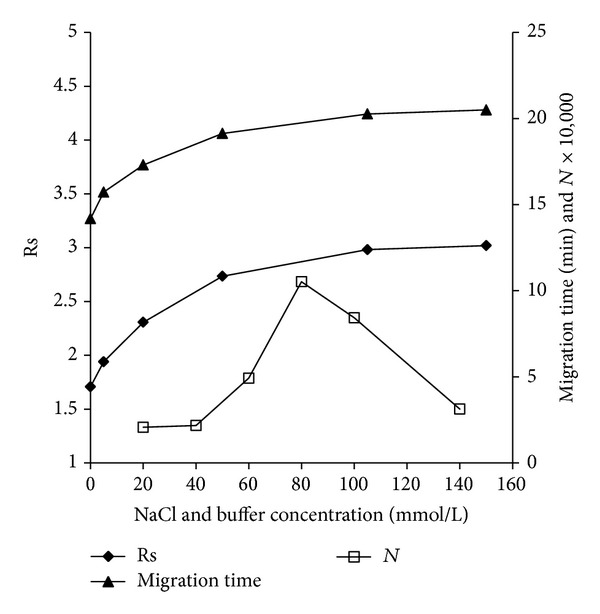
Influence of buffer concentrations on theoretical plate numbers, influences of NaCl concentration on Rs and migration times.

The influence of buffer concentration, ranged from 20.0 mmol/L to 140.0 mmol/L, on stacking was investigated (Figure 2). Theoretically, to some extent, higher buffer concentration provided larger conductivity difference between buffer and sample zone, which in turn might perform better primary stacking effect. In our works, stacking could not take place at the low buffer concentration (<40 mmol/L), as the conductance of the buffer was similar to that of the sample zone, the difference of migrating velocity of the MEL in buffer and in sample zone was not large enough to initiate stacking. When buffer concentration was higher than 40.0 mmol/L, stacking phenomena occurred. As the buffer concentration increased, the theoretical plate number (*N*) increased firstly followed by decreasing when the concentration was higher than 80.0 mmol/L, which may heat due to the broaden effects of extra Joule. Finally, a concentration of 80.0 mmol/L was selected for further studies.

### 3.2. Influence of Sample Matrix

**Figure 3:**
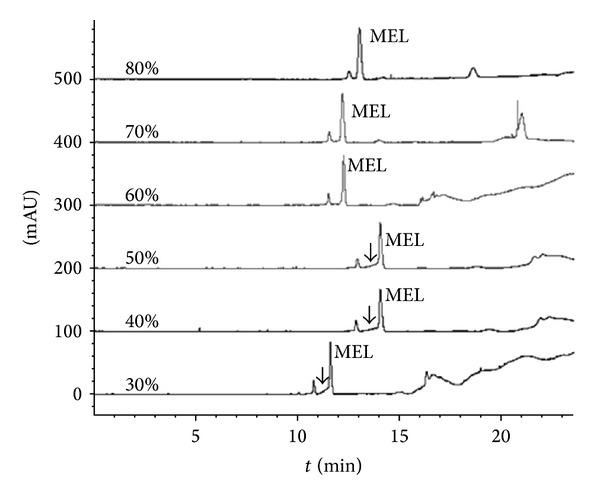
Electropherograms of milk treated with different percentages of acetonitrile. Condition: samples were treated with different percentages of acetonitrile (v/v) and were injected hydrodynamically at 50 mBar for 50 s. All samples contain 10 mmol/L NaCl and 8 mmol/L pH 2.8 phosphate solution and 3 *μ*mol/L MEL.

The effect of the acetonitrile concentration in sample on stacking was studied and optimized as organic reagent acted as accelerator for speeding up the velocities of analytes [15]. It could be seen (Figure 3), as the percent of the acetonitrile was lower than 50%, that the baseline around MEL became unsatisfied (arrow pointed in Figure 3) for separation and quantification, although the Rs value was higher enough between the interferent and MEL (Rs > 3.5). When the percentage was larger than 50%, baseline separation and stacking were both achieved. Meanwhile, the Rs decreased from 3.27 (60%) to 1.53 (80%) as the percentage of acetonitrile increased. Therefore, a percentage of 60% was considered as the better condition that provided less sample dilution and better Rs.

NaCl concentration was also an important factor that affected stacking, as the Na^+^ acted as leading ions in ASS procedure. Normally, almost all milk products contained Na^+^, and the [Na^+^] varied in larger range (from 0.18% to 1.5% in mass/volume form or in another form, 3~25 mmol/L). In this section, same real sample was added with different amount of NaCl (in range of 0~150 mmol/L) and was tested under same condition. The influences of NaCl concentration on Rs and migration times were shown in Figure 2. As the NaCl concentration increased, the migration time and Rs increased correspondingly (Rs ranged from 1.7 to 3.0, while migration times ranged from 14.2 min to 20.5 min) until the concentration of NaCl was higher than 100 mmol/L; the similar stacking and separation results were obtained. All these indicated that the method had a good tolerance on various concentrations of NaCl, which made it more suitable for assaying kinds of milk products. Finally, to keep the migration times similar, in the later assays, 100 mmol/L NaCl was added to the treated real milk samples before measurements.

### 3.3. Selection of Injection Times

**Figure 4:**
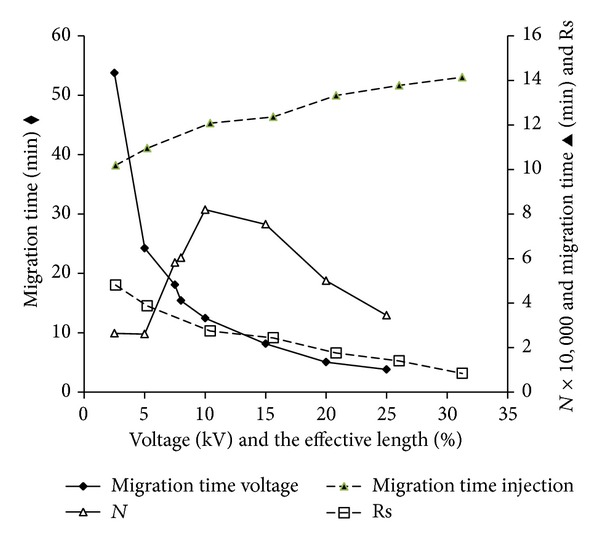
Influences of injection volume on migration times and Rs, effects of voltages on stacking.

As the injection time (presented as percent of the efficient length) was closely related to sensitivity of the method, the maximum injection time was carefully selected in this section.  Figure 4 showed that migration times increased (as a result of decreasing EOF) while the Rs values decreased (mainly caused by the decreasing efficient capillary length) with increasing injection times (similar to the results of Gao's work [25]). Based on the Rs value between MEL and interferent, 20% of the efficient capillary length (50 mBar × 40.0 s) was considered as a best injection percent (Rs = 1.75).

### 3.4. Effect of Stacking Voltage

In this section, stacking voltage was investigated in detail (Figure 4). It could be seen that* N* value appeared as a bell-shaped curve while the voltage increased from 2.5 kV to 25.0 kV. And the migration times decreased as the voltage increased. Experimental data showed that neither under lower nor higher voltage could MEL be well stacked. A voltage of +10.0 kV (ground at outlet) was finally decided to be the best condition as it was relatively less time-consuming and of highest* N* value (82,000).

### 3.5. Additional Rinsing Step

**Figure 5:**
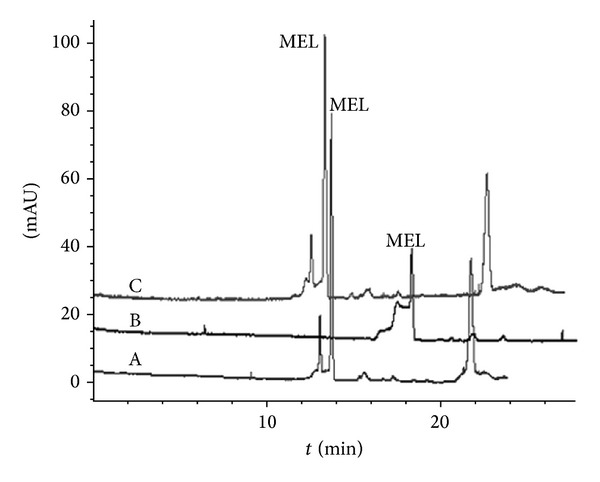
Electropherograms of real sample with/without the NaOH flushing step. (A) The first run of real sample with NaOH rinsing step; (B) the second run without NaOH rinsing step; and (C) the third run with NaOH re-rinsing. Condition: see Figure 3.

As the status of the inner-wall silanol greatly affected reproducibility of the method, special rinsing step was added before each run. It could be seen in Figure 5 that an additional 0.1 mmol/L NaOH rinsing step was crucial for maintaining reproducibility. Without NaOH rinsing step (capillary was only flushed with water and running buffer for 1.0 min and 3.0 min respectively, Figure 5-(B)), the separation and stacking were ruined compared with the first run (Figure 5-(A)). When the NaOH rinsing step was added before the next assay, better separation and stacking regained (Figure 5-(C)). For the selection of the NaOH rinsing time, our data proved that the 0.1 mmol/L NaOH rinsing time should be at least 3.0 min in order to achieve satisfied reproducibility.

### 3.6. Validation of the Method

Under the optimized condition, MEL could be easily detected within 20.0 min. Six replicates of the samples were used to test the inter- and intraday reproducibility of the method both for migration times and peak areas. Linearity range, using area as the function of the concentration, was obtained by adding different amount (final concentration of MEL ranged from 0.01 *μ*mol/L~50 *μ*mol/L at ten concentration levels) of MEL directly into untreated real milk samples. Recovery was tested with adding known amount of MEL to real samples under two final concentration levels (0.5 *μ*mol/L and 5.0 *μ*mol/L). LOD was calculated at signal/noise (S/N) = 3. All the related data were shown in Table 1.

### 3.7. Assay of Real Milk Samples

**Figure 6:**
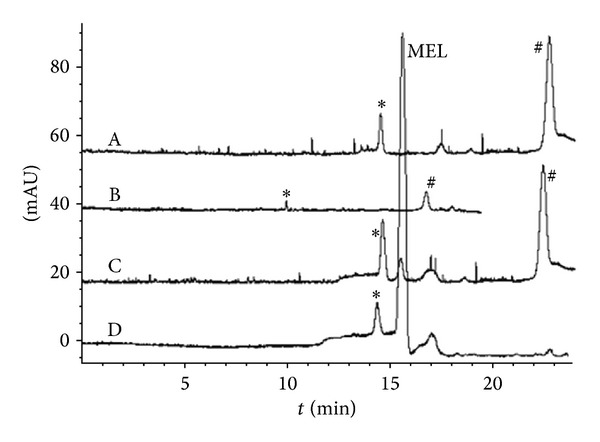
Electropherograms of real milk samples under optimized condition. (A)/(B)/(C) were samples from YILI pure milk; (D) was from WANGZAI reconstituted milk. Samples were treated with 60% acetonitrile (v/v) and were injected hydrodynamically at 50 mBar for 40 s. All samples also contained 8 mmol/L pH 2.8 phosphate solution and partial samples were added with MEL ((B)/(C) 0.5 *μ*mol/L; (D) 8 *μ*mol/L). Stacking was performed under +10.0 kV and results were detected at 200.0 nm.

The proposed method was applied to real milk samples. Separation and stacking results of two bands of milk products (YILI pure milk and WANGZAI reconstituted milk) were shown in Figure 6. It could be seen that MEL was well separated (from the interferent, ∗ in Figure 6), stacked, and detected in both tested milk samples using the established method within 20 min. For example, the blank YILI sample (without adding MEL, Figure 6-(A)) showed no MEL peak even under stacking mode, while a low concentration of 0.5 *μ*mol/L MEL could be easily detected (Figure 6-(C)) under stacking mode, with a ~20 folds enhancement of sensitivity compared with traditional CZE mode (Figure 6-(B)); for the assay of WANGZAI reconstituted milk (with adding MEL at a final concentration of 8.0 *μ*mol/L), similar phenomenon occurred (Figure 6-(D)). All these proved that this method was applicable for analyzing MEL in milk products.

## 4. Conclusion

An ASS method for separation and determination of melamine in milk products was proposed. Under the optimum conditions, low concentration of melamine (0.03 *μ*mol/L) in milk products could be successfully determined, which can cope with the maximum tolerable limits set worldwide for melamine and related compounds [26]. The established method also provided simple sample treatment steps, good reproducibility, and a wider linearity which indicated that it is suitable for routine assay of melamine in milk products.

## Figures and Tables

**Table 1:** Characters of established method.

	Intraday	Interday
Time (RSD%) (*n* = 6)	2.7	4.8
Area (RSD%) (*n* = 7)	2.2	4.9

Recovery (%)	0.5 *μ*mol/L	5 *μ*mol/L
	95%	112%

Linearity range (*μ*mol/L)	0.05~10	
Function and *R* ^2^	Area = 12.679 × Conc + 4.7555 *R* ^2^ = 0.9907	
LOD (*μ*mol/L)	0.03	
